# Preliminary investigation on stability and hydraulic performance of geotextile sand container breakwaters filled with sand and cement

**DOI:** 10.1038/s41598-022-19673-9

**Published:** 2022-09-09

**Authors:** Tom Elias, Kiran G. Shirlal

**Affiliations:** National Institute of Technology Karnataka, Surathkal, India

**Keywords:** Engineering, Civil engineering

## Abstract

Breakwaters are essential constructions providing tranquility to ports and harbour structures, when there is a lack of natural protection measures. Traditionally these massive structures are constructed using natural rocks weighing tonnes. In the present scenario, obtaining huge natural rocks are difficult as well as non-eco-friendly. Geotextiles sand containers (GSCs) emerges as a suitable alternative for the rock armour units of breakwaters and various literatures supports its efficacy. The present investigation aims at analysing the performance of GSCs when filled with a calculated amount of cement and sand. The Hydraulic performance and stability analysis of cement and sand filled geotextile breakwater models are carried out in a 1:30 scaled monochromatic wave flume. When GSC breakwaters are filled with sand and cement, up to 43% increased stability is observed with a considerable decrease in wave runup, rundown and reflection, than sand-alone filled units. As a result, cement-sand filled GSC units can be suggested as a possible alternative to sand alone filled units where vandalism has to be countered.

## Introduction

Breakwaters are generally constructed to dissipate wave energy, creating a calm condition inside the harbour area for easy loading and unloading of cargo from a berthed ship at a port. Additionally, a calm harbour area is advantageous for the smooth functioning of ports and harbours. Ocean waves often cause serious coastal erosion during monsoon season and can be catastrophic for the coastal ecosystem, livelihood and communities. Global climate change and associated sea-level rise can cause serious wave actions, inundations and coastal flooding in the adjoining coastal areas^1^. Recurring cyclones and associated storms surges are a matter of recent concern^2–4^. Therefore, it is inevitable to have protection structures that can reduce the adverse impact of damaging waves on coastal areas.

Coastal protection structures may generally be hard or soft. Sand bypassing, dune replenishment, vegetative protection, beach restoration, etc. are considered soft solutions^5,6^. Submerged and emerged breakwaters, dikes, seawalls, revetments and groins etc., are adopted as hard solutions^7,8^. Over the years, rubble mound or rock armoured structures were the most commonly adopted breakwater system^9^. The alarming increase in the cost of natural rock, reduced availability and prohibition of quarrying in many states demand viable alternatives to rock structures^10^. Over the years, there have been tremendous innovations in artificial armour units. Concrete Cubes, Tetrapods, Accropods, Dolos, Core Loc etc., are some notable armour units^9,11–15^. Research extended in designing innovative breakwater structures. Some notable contributions in this regard include semi and quarter-circular breakwater^16,17^, plate breakwater^18^, tandem breakwater^19^, floating pipe breakwaters^20,21^, pile breakwater^22,23^ etc. Along with these, geosynthetics are also widely used for various coastal engineering applications. Geosynthetics refers to a wide range of natural or artificial polymeric material than can be used for various civil engineering applications^24^. Geosynthetics include geotextiles, geomembranes, geogrids, etc. (used in civil engineering applications including road construction, waste management, slope protection etc.) with geotextiles being widely used for coastal engineering applications^25,26^. Coastal engineering applications of geotextiles include revetments^27^, embankments^28^, breakwater^29^, armour units of breakwaters^30,31^ and other coastal protection structures^8,12,32–35^. Geosynthetic sand containers (GSCs) are proved to possess various benefits over conventional rock constructions^36^. It can supply a wide range of uniformly sized armour units, which is very difficult in the case of rocks^37,38^. Cost per unit volume can be reduced when the size of containers is large, reducing construction time significantly. Fill ratio affects stability and shape due to interlocking and flexibility. It can also be stacked to steeper slopes when compared to conventional structures. Another attractive feature of the geotextile constructions is the insitu filling capability of the tube or containers with locally available materials, making the construction cost-effective and rapid^39^. Despite the fact that GSC units produce visual impact, the above-stated advantages make sand-filled geotextile units a viable alternative to the primary and secondary armour units of a conventional rubble mound breakwater. There have been fewer attempts to quantify the performance of geotextile units in a breakwater structure, motivating one to pursue the present study.

Physical experimentations conducted by Elias et al.^30^ investigates the efficacy of sand encapsulated geotextile containers as the armour units of breakwater structures. The study confirms the utility of geotextile sand containers (GSCs) up to a wave height of 3.96 m (prototype) when used as armour units of breakwaters. Various arrangements, including single layer, double layer, and slope parallel placement have been investigated, with double layer arrangement exhibiting up to 18% higher and slope parallel placement showing up to 11% lower stability than single layer placement. As described in the study, these GSC units are susceptible to damage that can be incidental, biological or vandalism. Incidental damages include boats making a direct hit or anchoring on the structure, fishing involving sharp tools tearing the geotextiles, leading to sand loss^40^. Driftwood and ice also cause incidental damages^29^. Rodents and rats nesting around the geotextile containers, damaging it, is grouped as biological damage^41^. Food waste encourages the colonisation of rats resulting in them tearing the geotextiles. Vandalism or deliberate destruction of GSC structures is a major concern and remains a prime disadvantage. It is reported that exposed GSC structures attract the curiosity of the native people or tourists and ultimately cut or damage it with sharp tools. Vandalism due to knife cut resulted in the failure of Kirra Groyne (Gold Coast, Australia), Submerged Reef at Kovalam, Kerala, St Clair revetment (Dunedin, New Zealand) etc.^29,41^. Once the containers are cut, the sand leaks out due to wave attack leading to total deflation and failure. As a possible solution for this problem, the present study investigates the feasibility of mortar mixture in filling the GSCs of breakwaters. When the GSC units are filled with mortar, the units harden, forming a rigid structure. This reduces the risk of vandalism as even when the external geotextile cover is damaged, the inner solidified unit remain intact.

GSC structures are often filled with sand. Dry sand, sand slurry (generally 70% water 30% sand) and even dredged materials are used to fill the containers^42^. Cement-sand mortar used in filling the containers are not very novel. Silvester^43^ carried out experiments in filling the geotextiles containers (sausage shaped) with cement and sand. Cement and sand mixture is considered in the form of a slurry with 100% slump value, allowing easy movement in any parts of the container. These containers, when hardened, should possess the strength of limestone. According to Silvester, the cost of structures made with grout filled sausages can be as low as 12% compared with limestone counterparts. Rajagopal et al.^44^ describe protection structures using geosynthetics at Pulickat Lake, Tamil Nadu, India. The breakwater structure constructed at the site is trialled with cement-sand mixture (10% cement). Cement sand mixture is filled into containers in dry form. When immersed in water, hardening takes place within 24 h, even in the marine environment. This helps in reducing the migration of sand inside the containers, thereby improving the performance. The authors point out the scope for quantifying structure behaviour when the percentage of cement is varied. Geo-mattresses are novel structures pumped with cement and sand slurry to form a mattress cover^45^. They are generally used in slope protection and river protection in estuarine areas. When cement and sand mortar is used as a filling material, geo-containers transform into a hard substance. Even when the fabric form is degraded, the hardened sand remains intact^43^, forming more ‘vandal-resistant’ units. All the above factors lead us to investigate the suitability of mortar filled GSC units as armour units of breakwaters. The present paper compiles the experimental observations and inferences and attempts to;

- Analyse the hydraulic performance of breakwaters armoured with cement and sand filled geotextile units.
- Analyse the stability and damages of breakwater structures armoured with cement and sand filled geotextile units.
- Compare the performance of cement-filled armour units with sand alone filled GSC breakwater structures.

## Physical modelling

### Wave flume

**Figure 1:**
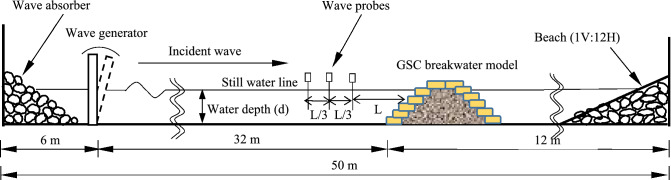
Schematic representation of GSC breakwater model at Wave Mechanics Laboratory, NITK^26^.

The physical model investigations were carried out in the monochromatic wave flume at the Department of Water Resources and Ocean Engineering, National Institute of Technology Karnataka (NITK), Surathkal, India, (Fig. 1). It is inferred from the findings of Faraci^46^ that there is no significant difference in the results of GSC structure wave response with monochromatic and random waves. Additionally, when experimenting with monochromatic waves, more conservative stability results may be obtained. Therefore, it has been decided to proceed with monochromatic waves (despite the fact that this is not the most cutting-edge technology available today). The available two-dimensional, fixed bed wave flume having a width of 0.74 m, a depth of 1.1 m, and a length of 50 m, including 25 m of glass panels for photography and viewing is used for this study. One end of the flume is provided with a passive wave absorber and the other end is facilitated with a bottom-hinged flap type wave generator that can produce waves. The flap movements are controlled by a 11 kW, 1450 rpm induction motor to create the waves. With the existing equipment, waves with periods ranging from 0.8 to 4 s and heights ranging from 0.02 to 0.20 m can be generated in a water depth of 0.50 m. For the current study, a model scale of 1:30 is used to confine Froude's Similitude criteria with the wave data of Mangaluru (Karnataka, India) coast.

### Instrumentation

The incident and reflected wave heights are measured using three capacitance type wave probes provided at the seaside, with a 0.001 m precision. Wave probes, an amplification unit, and a computer data collecting system make up the total instrumentation facility. The capacitance difference between water and copper conductor is measured by the wave probes. The laboratory wave recorder converts this digital voltage input into wave height and period. The measurement system's accuracy is judged to be around 3%. Wave runup and rundown are recorded using manual observation with calibrated strip charts pasted on the glass panels.

### Breakwater model construction

**Figure 2:**
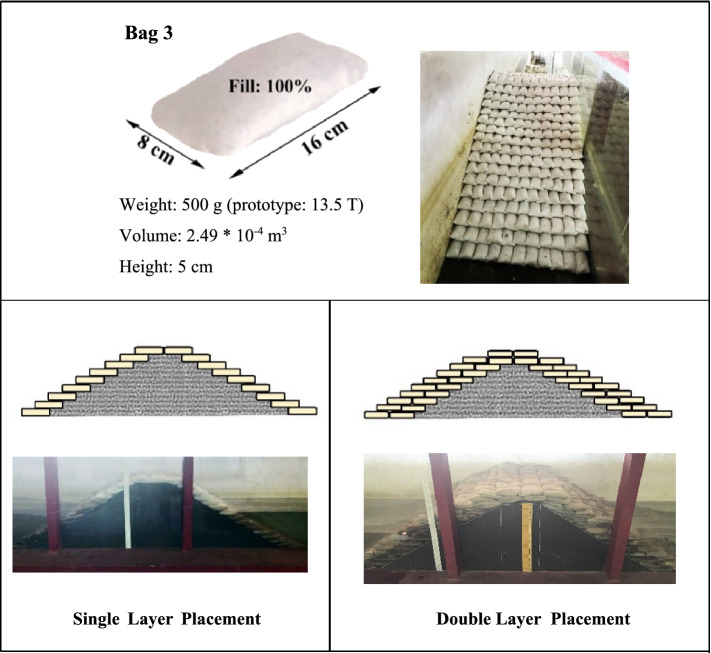
Dimensions and placement modes GSC units used in the model studies^30^.

The breakwater comprises a core made of m-sand and layers of GSC units filled with cement and sand. The core is to be made of quarry run in the prototype. The core is scaled using Froud’s criteria, and the grading is done as per the previous studies conducted^47^ in the same wave mechanics laboratory. The core grading resulted in fine particles due to the selected scale, making it impermeable in the model scale. The elaborate structural design of GSC breakwater is discussed in Elias et al.^30^ and is beyond the scope of the current paper. Geotextile scaling is almost unattainable due to the difficulty of fabricating 30 times thinner fabric. Details of properties and governing parameters of the breakwater model are provided in Tables 1, 2, 3. As a preliminary investigation, the emerged, non-overtopping breakwater model is tested with a single layer of cement-sand filled geotextile armour units (see Fig. 2). As inferred from the work of Rajagopal et al.^44^, the cement percentage to be added to GSCs is estimated to be 15 and 20% of GSC weight. The individual GSC units size, volume, shape, arrangement etc., are selected based on the extensive experimentations by Elias et al.^26,30^. Equation (1) is used for the volume calculation of a fully inflated rectangular bag with length a and breadth b, which cannot stretch or shear^48^

1 $$V = a^{3} \left[ {\frac{b}{\pi a} - 0.142\left( {1 - 10^{{\frac{ - b}{a}}} } \right)} \right]$$

**Table 1:** Range of governing variables.

Variable	Expression	Range
Wave height (m)	H	0.06, 0.08, 0.10,0.12, 0.14,0.16
Wave period (s)	T	1.2, 1.4, 1.6, 1.8, 2, 2.2
Water depth (m)	d	0.35, 0.40, 0.45
Angle of attack	F	90°
Mass density (GSC) (kg/m^3^)	Ρ	2005
GSC armour weight (g)	W	500
Crest height (m)	H	0.70
Crest width (m)	B	0.32, 0.29
GSC material		Non-woven

**Table 2:** Non-dimensional model and wave characteristics.

Variable	Range
**GSC breakwater model characteristics**	
Slope	1 V:2H
Relative height (h/d)	1.55–2
Relative crest width (B/d)	0.644–0.91
Relative water depth (d/gT^2^)	0.007–0.023
**Wave characteristics**	
Wave steepness(H_0_/gT^2^)	0.00126–0.0083
Surf similarity parameter (tanα/(H_0_/L_0_)^0.5^)	2.18–5.68

**Table 3:** Properties of construction materials used.

Property	Range
**a. Geotextiles**	
Type	Non-woven
Material	Polypropylene
Colour	White
Mass (GSM)	200
Tensile strength (kN/m)	12
Elongation at max tensile strength	30%
Permeability (m/s)	6 * 10^–2^
Thickness (mm)	1.2
Apparent opening size (mm)	0.2
**b. Sand**	
Location	NITK Beach
Specific gravity	2.65
D_10_ (mm)	0.18
Median grain size D_50_ (mm)	0.35
**c. Core**	
Material	M-sand
Specific gravity	2.78
D_10_ (mm)	0.22
D_50_ (mm)	0.45

The study examined four sandbags, namely, Bag 1, Bag 2, Bag 3 and Bag 4, varying in size and sand fill percentage. Bag 3 of 0.16 m length and 0.08 m width is found to be the best performing armour unit with regard to hydraulic performance and stability; therefore, the present investigation is limited to experiments on Bag 3, which would be nearly 13.5 tonnes in the prototype. The construction can be carried out using similar methods of conventional rubble mound breakwaters. Placing of GSC units can be carried out using split bottom barges, GPS positioning etc. Resistance of the bags in the field will be more than what is experienced in the model due to scale effects. The units are stitched after filling with the calculated amount of cement and sand in dry form. The units are then cured for up to 24 h. Hardened units are arranged over the breakwater core with their longer dimensions parallel to wave attack (based on the findings of Shirlal and Mallidi^49^). After the model is completely constructed, water is pumped to the desired depth in the wave flume.

### Methodology

**Figure 3:**
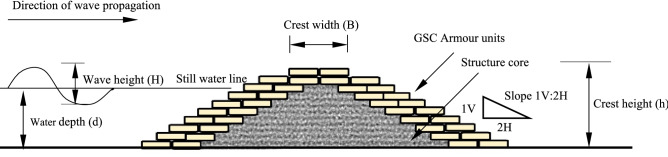
Representation of the constructed GSC breakwater model^26^.

The constructed model is exposed to selected wave climate in order to assess the hydraulic performance of the GSC breakwater, which includes wave runup, rundown and reflection. The structure is initially subjected to smaller waves of 0.06 m, which are gradually increased up to 0.16 m at an interval of 0.02 m for a set wave period. Waves reflected from the structure can reach the wave generator, changing the desired wave conditions. To avoid such problems, wave attack will be limited to a burst of no more than eight waves, with the generator shutting down allowing brief gaps between each test case to dampen the wave energy and create a still water surface. Additionally, wave absorbers at beach end and generator end are provided confining to the findings of various literature^50–52^. Wave responses like runup, rundown and reflection are calculated for each condition (for a fixed wave height and period for a water depth) by sending three sets of wave trains comprising eight waves each. Isaacson^53^ proposed a three-probe approach for computing the reflection coefficient K_r_. Three wave probes are placed seaward of the breakwater model at a distance of L and L/3. K_r_ is calculated using the wave amplitudes obtained at the probes. Wave amplitudes from three probes are observed for an intended wave height, and period is supplied to the formulations proposed by Isaacson. The reflection coefficient changes in time due to wave-wave interaction and possible breakwater deformation. Being a derived parameter, reflection coefficient calculated using the stated method is expected to show up to 20% error. The strip charts pasted on the glass panels of the flume are used to record the runup and rundown values. Runup and rundown are the maximum vertical limits of wave uprush and down rush on the structure, measured with respect to still water level. Since a burst of mere 8 waves are used and no sophisticated instruments involved in recording these quantities, the wave runup and rundown values obtained need not be accurate. Figure 1 depicts the experimental setup while Fig. 3 gives the design parameters of the breakwater model.

Unlike conventional rubble mound breakwaters, stability and damage classification of GSC breakwaters are carried out in a unique way, as suggested by Dassanayake and Oumeraci^54^. The breakwater structure is exposed to waves of desired height and period, and the wave-structure interaction is analysed. Armour unit displacement/movement and detachment ar**e** inspected and recorded, and then categorised into damage categories DC0 through DC4, as shown in Table 4. Armour units can be stable, displaced or detached according to the criteria given in Table 4. Displacement and detachment of individual units are quantified using manual examination. The current structure is exposed to up to 3000 waves to define damage levels comparable to 6–11 h of actual storm duration^55^. Suppose the GSC units exhibit ‘stable’ condition (according to Table 4) for the entire storm duration; the configuration is classified as DC0 (“No Damage”) for the particular wave height and period. Experimentation proceeds by increasing wave height and period and classifies the structure into various degrees of damage. Before moving on to the next case, the damaged structure is reorganised. Water is drained from the flume, and the core is reassembled for testing with a new configuration of GSC units, once the configuration has been explored for all test wave conditions. In case of mortar units, waves will cause pulsating and impact forces when the units are rocking, leading to fatigue. As the number of cycles of wave loading increases, initiation and propagation of cracks occurs resulting in the breaking of units. Limiting wave attack to a burst of 8 waves helps in reducing the effect of cyclic lading so that progressing waves are not creating fatigue to GSC units by the time the wave responses are recorded.

**Table 4:** Damage classification^54^.

Damage classification I (single GSC) Considering only a single GSC in the most vulnerable position (critical GSCs)				
“Stable”	Horizontal displacement < 10% of GSC length (or width)/upward rotation < 10^0^			
“Movement”	10% of GSC length (or width) < Horizontal displacement < 50% of GSC length (or width)			
“Detachment”	Horizontal displacement > 50% of GSC length, width, Upward rotation > 45^0^			

Damage classification II (GSC-structure) Considering all critical GSC layers of GSC-structure				
No damage [DC0]	Incipient motion [DC1]	Minor damage [DC2]	Medium damage [DC3]	Total failure [DC4]
< 10% of critical GSCs moved No critical GSCs detached	10–50% of critical GSCs moved < 5% of critical GSCs detached	> 50% of critical GSCs moved 5–20% of critical GSCs detached	20–40% of critical GSCs detached	> 40% of critical GSCs detached

### Assumptions in model studies

Since actual field conditions cannot consistently be replicated in flume experiments, modelling a coastal structure necessitates some simplifications or assumptions. As a result, the current investigations are based on the following assumptions.

- The seabed is fixed and horizontal, therefore the experimentation is unaffected by sediment movement.
- Toe scoring resulting from sediment washout is not considered.
- The scaling of geotextiles and sand is not attempted and its influence on structure performance is not considered.
- The difference in density between seawater and freshwater (used in the wave flume) is not taken into account.

## Results and discussion

### Background

This section details the results of investigations carried out on geotextile breakwaters filled with mortar. From the previous experiments, Bag 3 is found to be the best performing model^26,30^. This best performing model of Bag 3 is then filled with mortar to analyse its efficacy when used as armour units of breakwater structure. In this context, the following configurations are tested in the wave flume;

1. Bag 3 filled with 15% cement, stacked in single layer.
2. Bag 3 filled with 20% cement, stacked in single layer.
3. Bag 3 filled with 20% cement, stacked in double layers.

#### Wave runup studies

**Figure 4:**
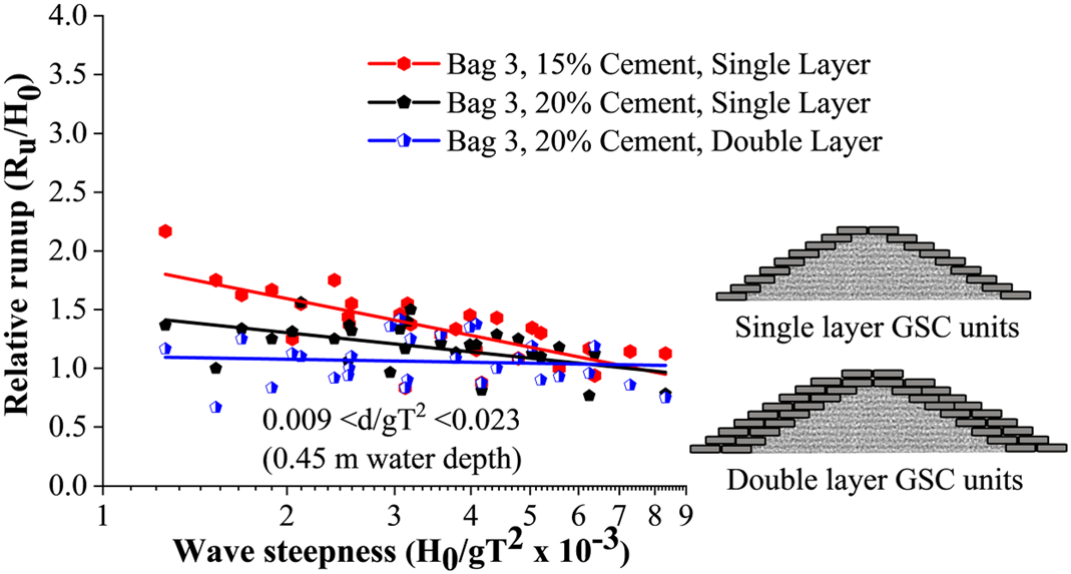
Relative runup of all tested configurations.

Wave runup height is recorded to calculate the relative runup height (R_u_/H_0_), which is beneficial for understanding the overtopping and transmission of water over the crest of the structure. Relative runup of all tested breakwater configurations is represented in Fig. 4. This shows that breakwaters of single-layer Bag 3 with 15% cement exhibited the maximum relative runup, with double layer 20% cement-filled configuration being the least. Breakwater with single-layer 15% cement filled configuration exhibited a maximum of 15.9 and 33.33% higher relative runup with respect to those with single layer of 20% and double layer of 20% cement bags, respectively.

**Figure 5:**
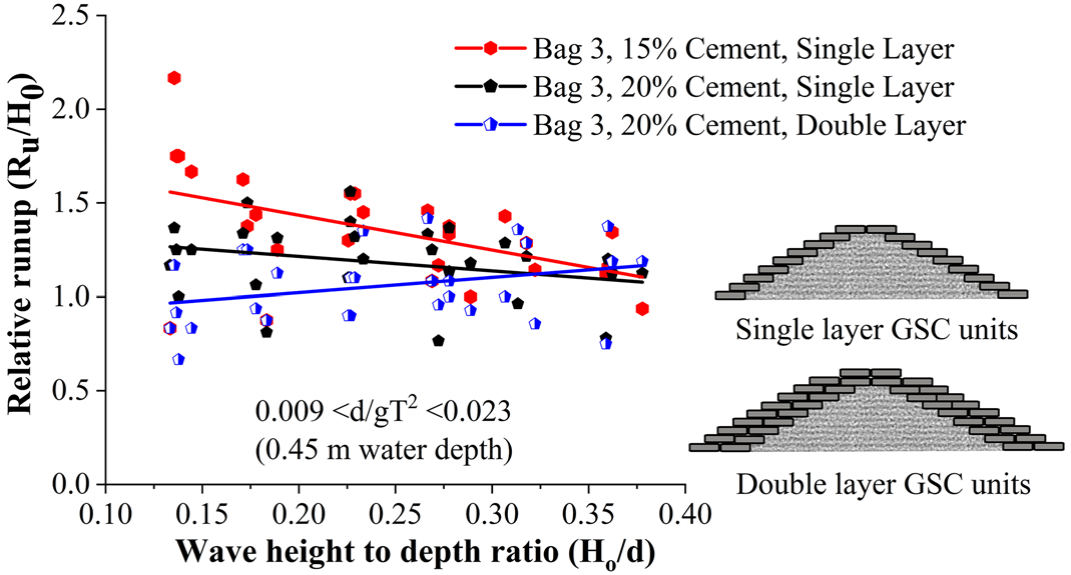
Relative runup vs wave height to depth ratio (H_0_/d) of all the tested configurations.

Relative runup vs wave height to depth ratio (H_0_/d) of all the tested breakwater configurations are represented in Fig. 5. This shows that single-layer breakwaters with 15% cement filled armour units had the maximum relative runup, with double layer 20% configuration being the least. This plot is helpful in analysing the efficacy of various placement modes.

Breakwaters armoured with 15% cement-filled bags showed a tendency to break within. Those broken bags tend to readjust in the structure slope resulting in the closure of certain pore spaces. As a result, wave dissipation reduced, leading to increased runup. When the bags were filled with 20% cement, they acted like solid units, creating more pore spaces. Those solid units provided no scope of readjustments, as a result, the pore spaces were not affected. This promoted increased wave dissipation on the structure slope resulting in the reduced runup. Further, when an additional layer was provided, the porosity of the structure increased, resulting in further wave dissipation on the structure slope. This resulted in the reduced runup rates of double-layer arrangement.

#### Wave rundown studies

**Figure 6:**
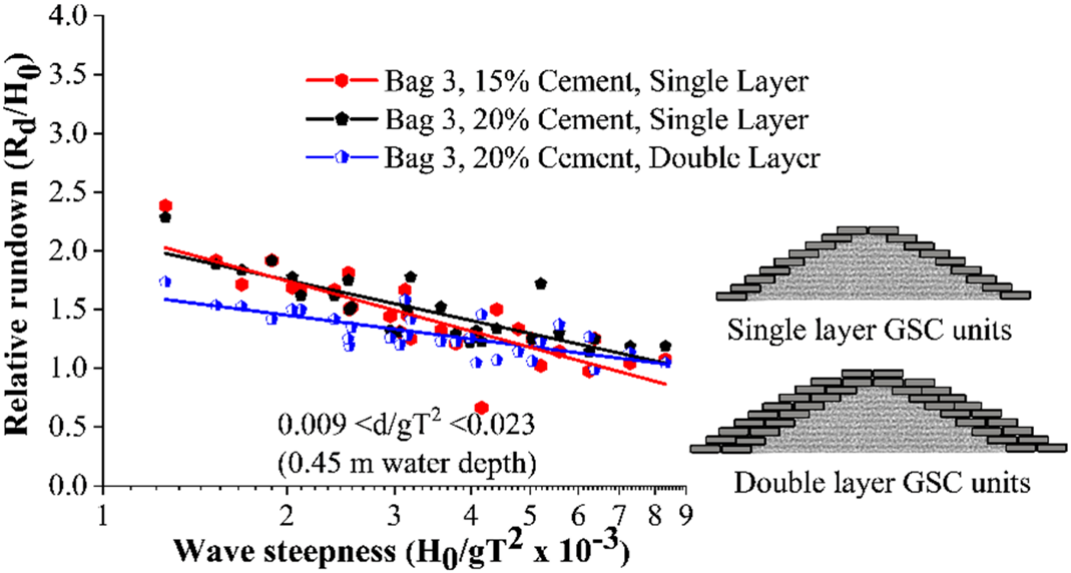
Relative rundown of all tested configurations.

**Figure 7:**
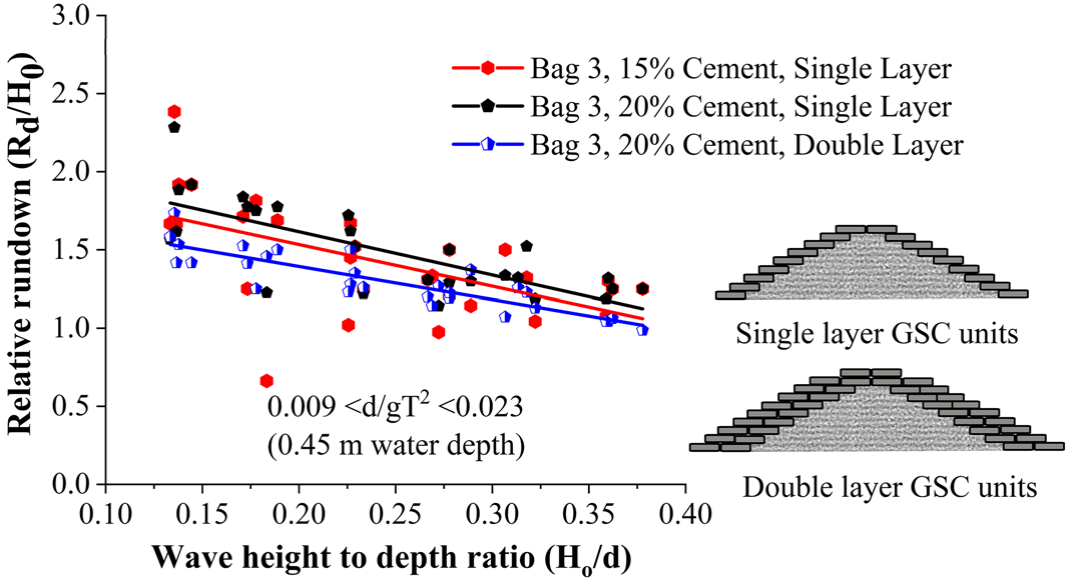
Relative rundown vs wave height to depth ratio (H_0_/d).

Wave rundown is helpful in understating the behaviour of water retreating from the structure surface. The relative rundown of all tested configurations is represented in Fig. 6. This shows that single-layer breakwaters armoured with 15% cement showed the maximum relative rundown, with double-layer breakwater structure of 20% cement-filled units being the least. Bag 3, 15% cement-filled single layer configuration represented 5 and 31.25% higher relative rundown with respect to single layer 20% (cement fill) and double layer 20% (cement fill) arrangements respectively. The relative rundown vs wave height to depth ratio (H_0_/d) of all the tested configurations is represented in Fig. 7. This shows that breakwaters armoured with single-layer 15% cement fill, showed the maximum relative rundown, with double layer 20% cement filled configuration being the least. This plot is useful in analysing the efficacy of various placement modes.

Single layer placements nearly represent a similar rundown behaviour, a maximum of 5% variation. When the armour units are arranged in double layers, increased porosity helped in wave dissipation, as a result, wave rundown decreased for double-layer structures.

#### Reflection analysis

**Figure 8:**
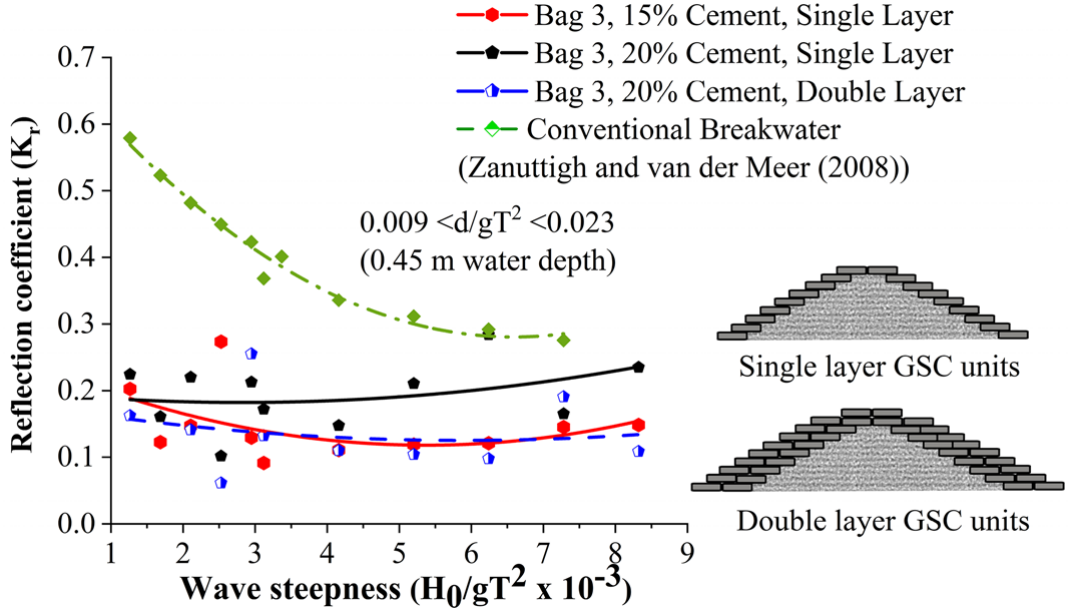
Calculated K_r_ values for all the tested cases.

Analysis of wave reflection from the breakwater are presented in this section. The three probes method by Isaacson^53^ is adopted in the estimation of reflection coefficient K_r_. The variation of K_r_ with wave steepness parameter for all the test cases is represented in Fig. 8. It is evident that all the test cases exhibited lower K_r_ values compared with convention breakwaters, as appeared in Zanuttigh and van der Meer^14^. Breakwater structure with single-layer configuration with 20% cement fill showed the maximum reflection, up to 76% higher than other models. The fitted curves exhibit a poor correlation coefficient, which is just up to 19%. Decreased K_r_ values are a result of increased porosity and consequent increase in wave dissipation on the structure slope.

#### Stability and damage analysis

**Figure 9:**
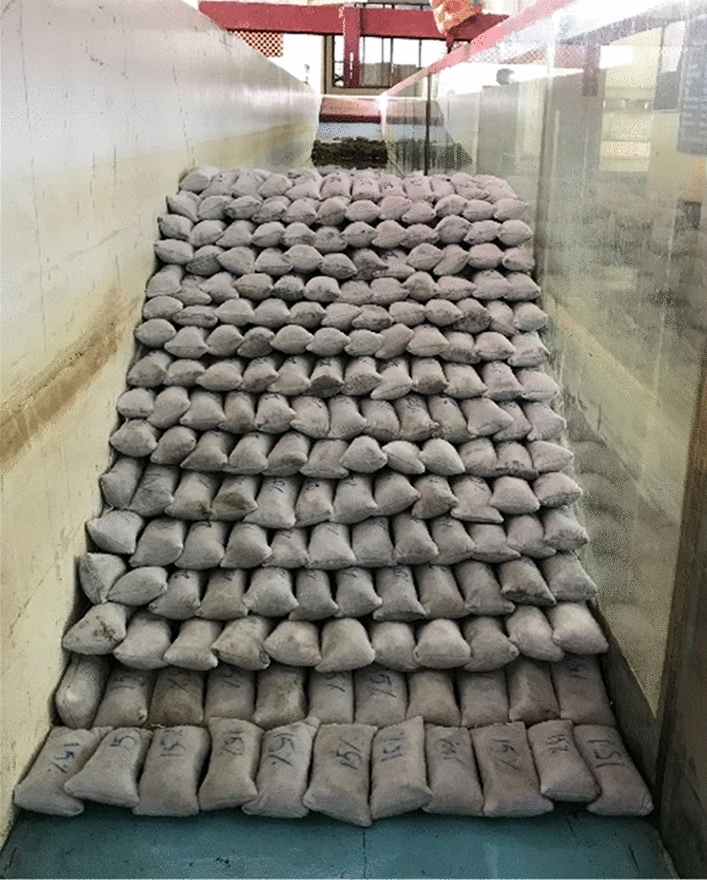
Image showing sand container filled with 15% cement and arranged to a single layer. Note the bulkiness of the units contributing to higher porosity.

It has to be noted that when the geotextile containers are filled with mortar, the resulting breakwater structure produces more pore spaces than containers with sand alone (Fig. 9). Internal displacement of sand, when subjected to wave action, has been stopped due to the cementation process. Additionally, the hard units tend to be comparatively stable than its sand alone counterparts. In most cases, pull-out was observed as the major failure mode. The process of pulling out of containers occur due to wave attack on armour units. According to Recio^56^, interface friction is the major factor controlling the pullout of GSC units. The friction properties of the material and contact area between two containers control the interface friction. In the case of cement-filled bag, the bags become bulky after solidification reducing the contact area between two containers, as a result, interface friction reduces, leading in increased pullout of GSC armour units.

As reported by Dassanayake and Oumeraci^54^ and Elias et al.^30^, incipient motion (DC1) curves can serve as a suitable tool to demarcate the stability of a particular case. C_w_ value is calculated for each incipient motion points Eq. (2) and its average value is used in (3) for drawing the stability curves.

2 $${\text{C}}_{{\text{w}}} {\text{ = N}}_{{\text{s}}} \cdot \sqrt {{\upxi }_{{0}} }$$

3 $${\text{N}}_{{\text{s}}} { = }\frac{{{\text{C}}_{{\text{w}}} }}{{\sqrt {{\upxi }_{{0}} } }}$$

where N_s_ is the stability number and ξ_0_ is the surf similarity parameter and is represented by (4) and (5). H_s_ is the incident significant wave height, ρ_w_ and ρ_GSC_ correspond to the density of seawater and GSC. D is the thickness of armour layer given by l.sinα, where l is the length of GSC armour units, α is the slope angle of the geosynthetic structure, L_0_ is the deepwater wavelength, equals to gT^2^/2π, where T is the wave period.

4 $$N_{s} = \frac{{H_{s} }}{{(\frac{{\rho_{GSC} }}{{\rho_{w} }} - 1) \cdot D}}$$

5 $$\xi_{0} = \frac{\tan \alpha }{{\sqrt {\frac{{H_{0} }}{{L_{0} }}} }}$$

**Figure 10:**
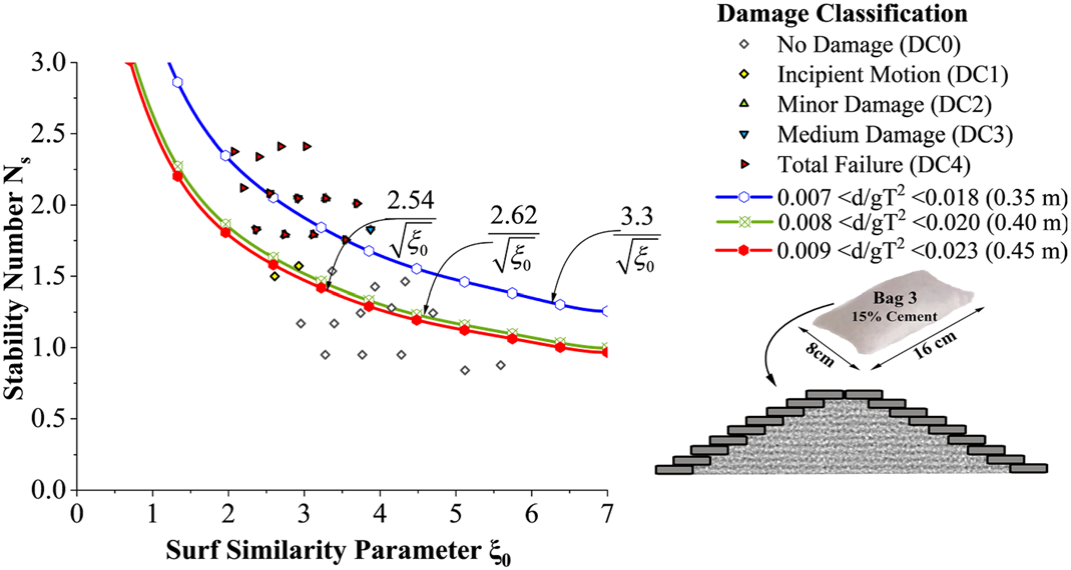
Incipient motion curves for Bag 3 filled with 15% cement, arranged to a single layer.

Incipient motion curves are obtained for Bag 3 filled to 15% cement, arranged to a single layer as shown in Fig. 10. Experimentations are carried out at three different relative water depths 0.007 < d/gT^2^ < 0.018 (0.35 m), 0.008 < d/gT^2^ < 0.020 (0.40 m) and 0.009 < d/gT^2^ < 0.023 (0.45 m). As seen from the graph, stability at lowest water depth (0.35 m) was found to be 25 to 29% higher than at 0.40 and 0.45 m water depths respectively. Higher wave activity, wave energy at increased depth of water can be the possible reason for this. Moreover, wave runup and rundown will be higher at higher depths of water resulting in increased instability.

**Figure 11:**
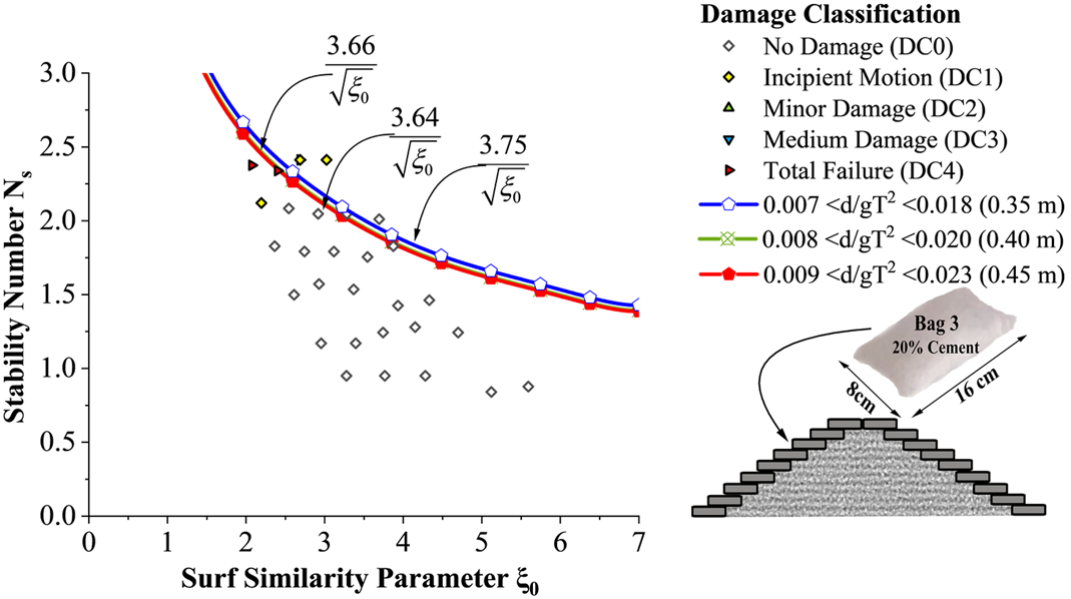
Incipient motion curves for Bag 3 filled with 20% cement, arranged to a single layer.

Incipient motion curves are obtained for Bag 3 filled to 20% cement, arranged to a single layer in Fig. 11. Experimentations are carried out at three different relative water depths 0.007 < d/gT^2^ < 0.018 (0.35 m), 0.008 < d/gT^2^ < 0.020 (0.40 m) and 0.009 < d/gT^2^ < 0.023 (0.45 m). as seen from the graph, stability at lowest water depth (0.35 m) was found to be 2.4 to 3.5% higher than at 0.40 and 0.45 m water depths respectively. Wave runup was smaller at 0.35 m water depth thus deformations occurred during runup will be comparatively lower.

**Figure 12:**
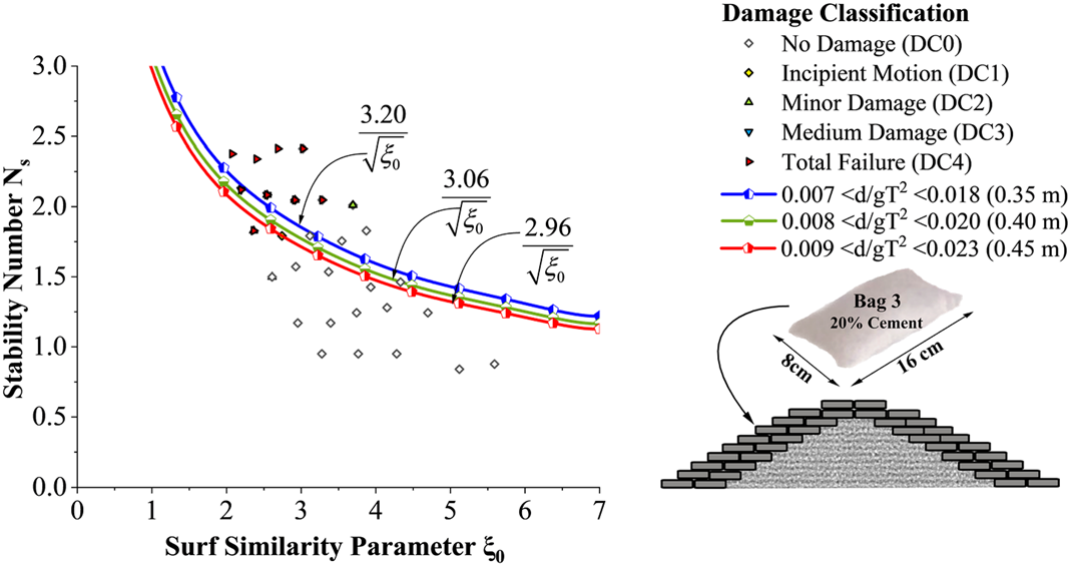
Incipient motion curves for Bag 3 filled with 20% cement, double layer.

But the deviation of stability is found to be comparatively lesser than 15% cement-filled case. Moreover, when the cement content in bags increased from 15 to 20%, the stability of the structure shot up to a maximum of 43.3%. Incipient motion curves obtained for Bag 3 filled with 20% cement, arranged in double layers are shown in Fig. 12. Experimentations are carried out at three different relative water depths 0.007 < d/gT^2^ < 0.018 (0.35 m), 0.008 < d/gT^2^ < 0.020 (0.40 m) and 0.009 < d/gT^2^ < 0.023 (0.45 m). as seen from the graph, stability at lowest water depth (0.35 m) was found to be 4.5 to 8.1% higher than at 0.40 and 0.45 m water depths respectively. Higher wave activity and wave energy at deeper depths may be the possible reason. Moreover, when the number of layers increased from one to two, a considerable decrease in stability, up to 23.6%, is exhibited. The major reasons behind these observations will be discussed in the subsequent sections.

As far as the hydraulic performance and stability are considered, breakwater structures with GSC armour units filled with 20% cement are observed to perform better than those units filled with 15% cement. Therefore, studies on structures with double layer GSC units filled with 15% cement are not carried out in the present study.

##### Effect of the number of layers and cement percentage

**Figure 13:**
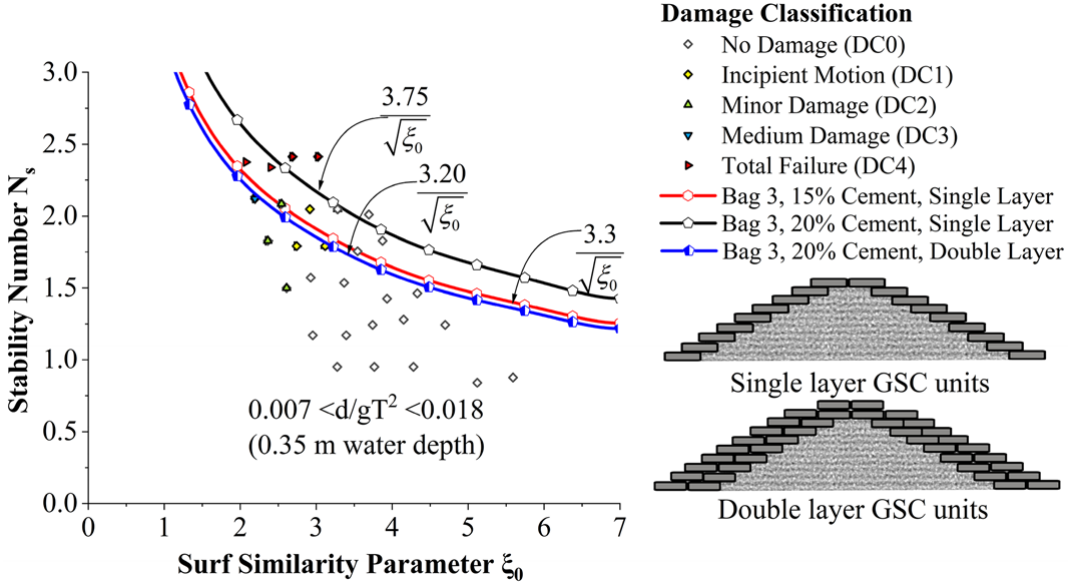
Stability curves of all experimented configurations for a relative water depth 0.007 < d/gT^2^ < 0.018 (0.35 m actual water depth in flume).

The effect of the number of layers and cement percentage has been tested in this section. Stability curves of all experimented configurations are analysed for a relative water depth 0.007 < d/gT^2^ < 0.018 (0.35 m actual water depth in flume) in Fig. 13. 20% cement-filled bags arranged in single layer exhibited increased stability, 13.63 and 17.18% higher than 15% cement single layer and 20% cement double-layer configurations, respectively.

**Figure 14:**
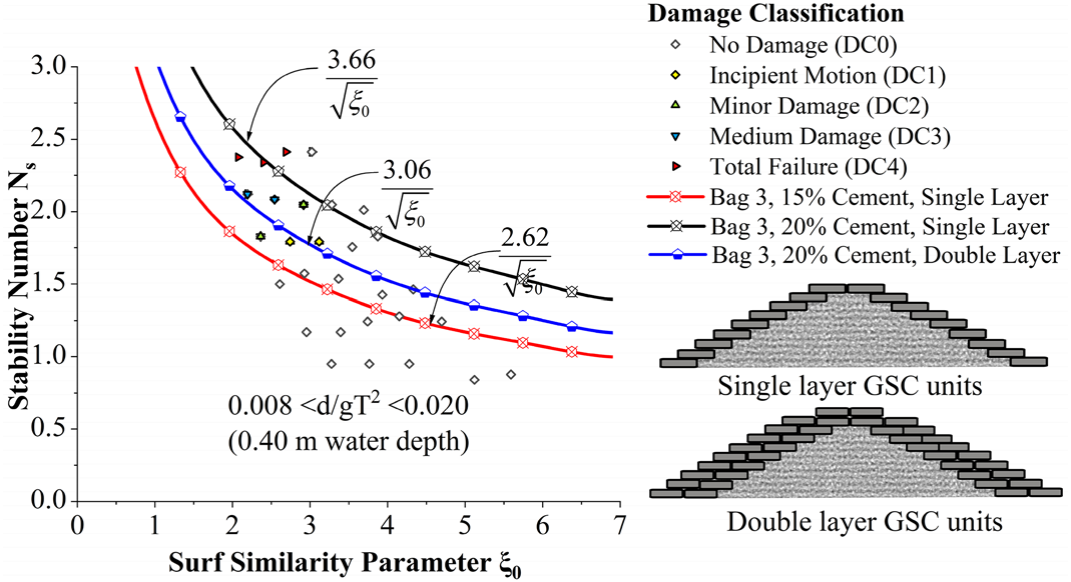
Stability curves of all experimented configurations for a relative water depth of 0.008 < d/gT^2^ < 0.020 (0.40 m actual water depth in flume).

**Figure 15:**
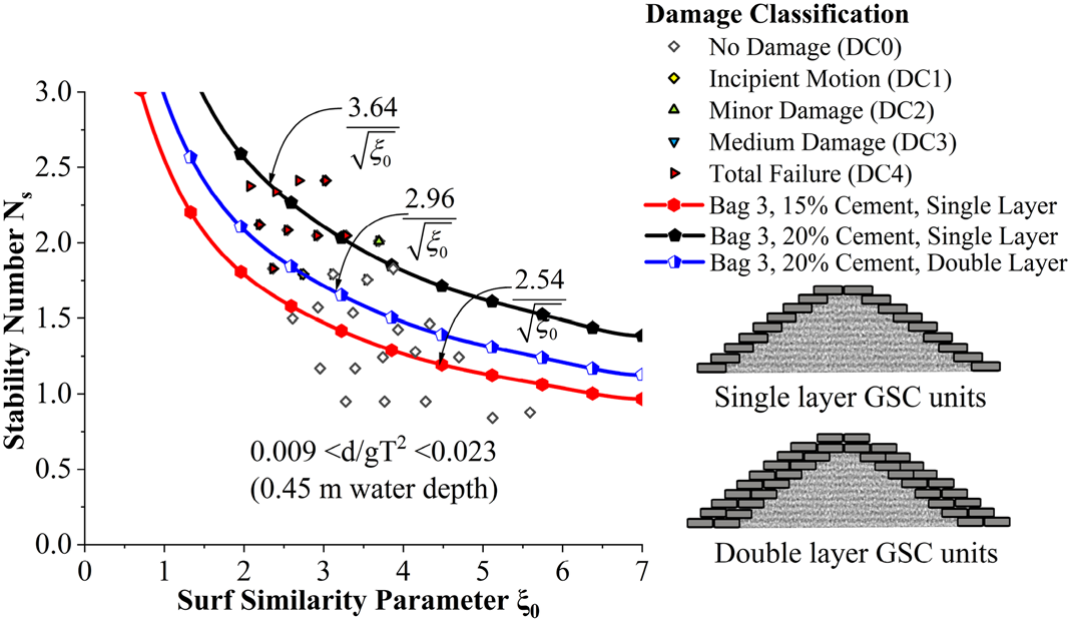
Stability curves of all experimented configurations for a relative water depth 0.009 < d/gT^2^ < 0.023 (0.45 m actual water depth in flume).

Stability curves of all experimented configurations are analysed for a relative water depth 0.008 < d/gT^2^ < 0.020 (0.40 m actual water depth in flume) in Fig. 14. 20% cement-filled bags arranged to single layer exhibited the highest stability, 39.6 and 19.6% higher than 15% cement single layer and 20% cement double-layer configurations, respectively. Stability curves of all experimented configurations are analysed for a relative water depth 0.009 < d/gT^2^ < 0.023 (0.45 m actual water depth in flume) in Fig. 15. 20% cement-filled bags arranged in a single layer exhibited the highest stability, 43.3 and 22.9% higher than 15% cement single layer and 20% cement double-layer configurations, respectively.

From the above analysis, it has been observed that when cement content is increased from 15 to 20%, there is a considerable increase in stability, i.e. 13.6 to 43.3%. Sand bag structure with 20% cement content exhibited higher strength. The strength of the bag increased because of an increased percentage of cement in it. This may be the reason as it could withstand higher wave activity. However, the hardened material inside certain 15% cement-filled bags showed a tendency to break within the container, making it vulnerable to detachment on higher wave activity.

**Figure 16:**
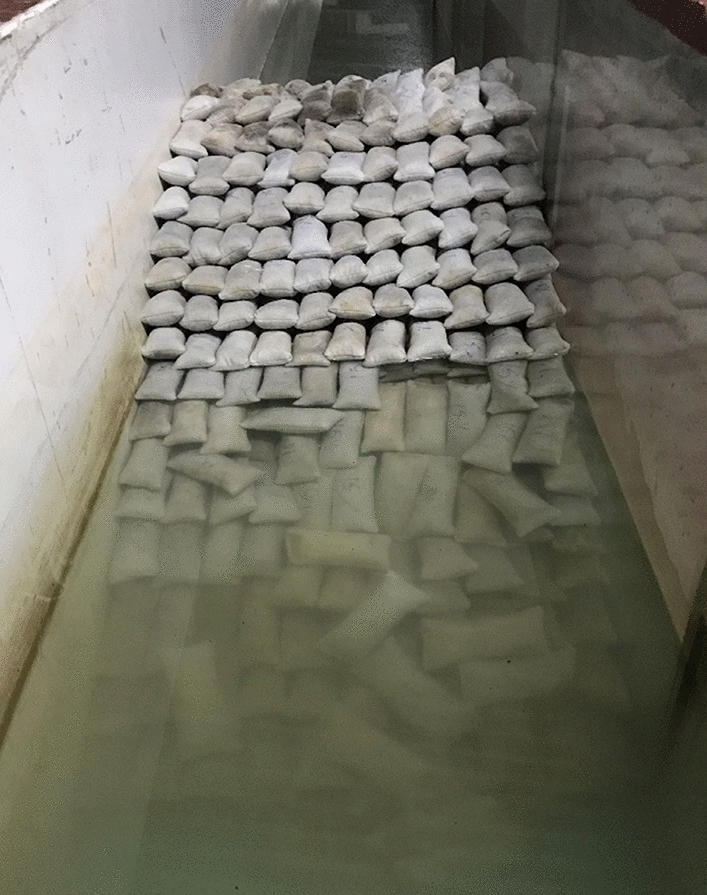
Image showing detached GSC units from the outer layer of breakwater model.

Similarly, when 20% cement-filled bags are altered from single layer to double layer, 17.18 to 22.9% decrease in stability is observed. This observation contradicted the general concept that stability increases when the number of outer armour layers increases. The reduction in stability of double-layer structure may be mainly attributed to the lack of friction experienced by the outer armour layer. As a result, units from the outer layer tend to get detached at lower wave heights as shown in Fig. 16. Bags with broken units inside practically looked the same when photographed. A case of armour deformation has been added and shown in Fig. 16.

### Comparison of cement and sand-filled configurations with other configurations

#### Comparative analysis of wave runup

**Figure 17:**
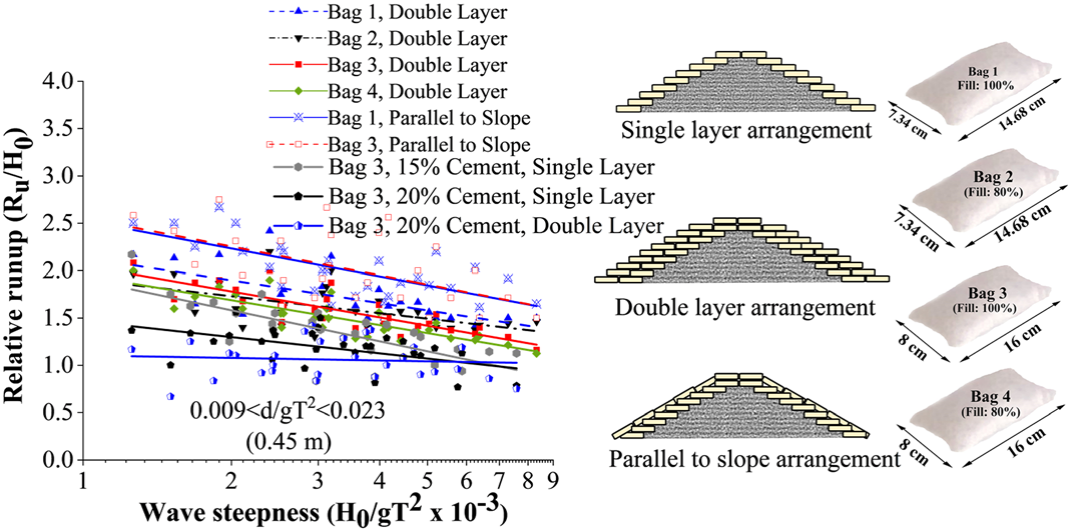
Comparative analysis of runup behaviour of all the tested configurations.

**Figure 18:**
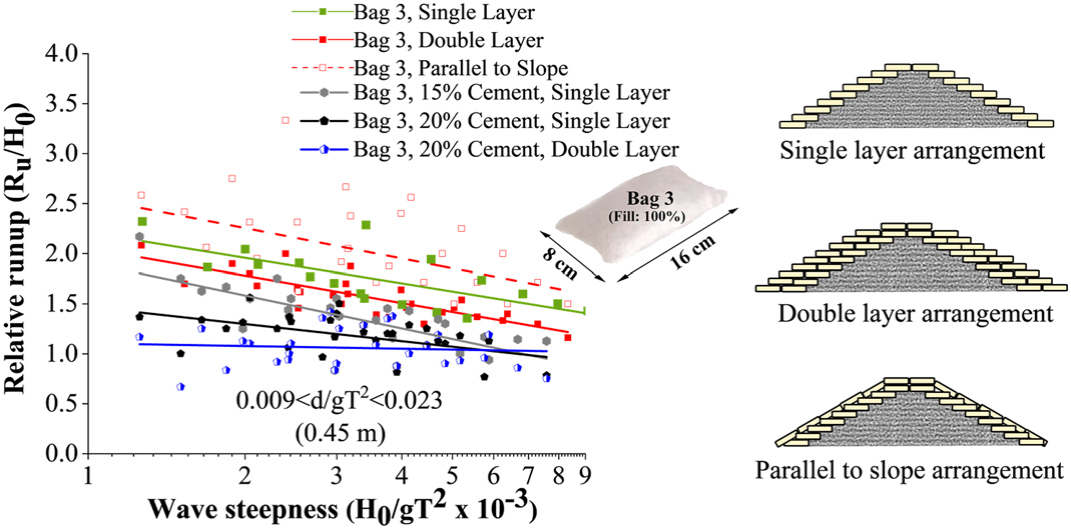
Relative wave runup for various placement modes of Bag 3.

Figure 17 shows the comparative analysis of the runup behaviour of cement-filled GSC armour units with other basis GSC armour configurations as discussed in Elias et al.^30^. The runup curves tend to vary from 0.5 to 2.7 on a scale of relative runup rate (R_u_/H_0_), of double-layer configuration of Bag 3 armour (20% cement-filled) showing the least runup rates and parallelly placed configuration showing the highest runup rates. Out of all tested configurations, runup rates are the lowest for cement-filled arrangements. This is due to the increased wave dissipation over the structure slope resulting in reduced runup rates because of higher structure porosity. Cement-sand filled structures experience higher porosity as there is no scope for readjustments so that the pores can be covered, as in the case of GSCs. Closer observations in Fig. 18 reveal that relative wave runup is considerably reduced as the number of layers increases from one to two. Single-layer Bag 3 structure with armour filled with sand alone exhibited a 13.15% to 8% reduction in runup rates compared with the double-layer arrangement. Similarly, up to 31% reduction is experienced in the runup rates on Bag 3 cement-sand filled double-layer configuration compared to its single layer counterpart. As the layer increases, more pore spaces are created, accelerating wave dissipation over the structure, thus reducing runup.

As far as placement method is compared, slope-parallel placement is observed to possess higher runup rates. Slope parallel placement exhibited up to 31.5% and 16.27% increased relative runup compared to double and single-layer structures. The main reason behind this observation is the continuous covering mechanism in slope-parallel placement, reducing the porosity of the structure. As a result, runup increases and more wave energy gets reflected due to its inability to dissipate energy.

#### Comparative analysis of wave rundown

**Figure 19:**
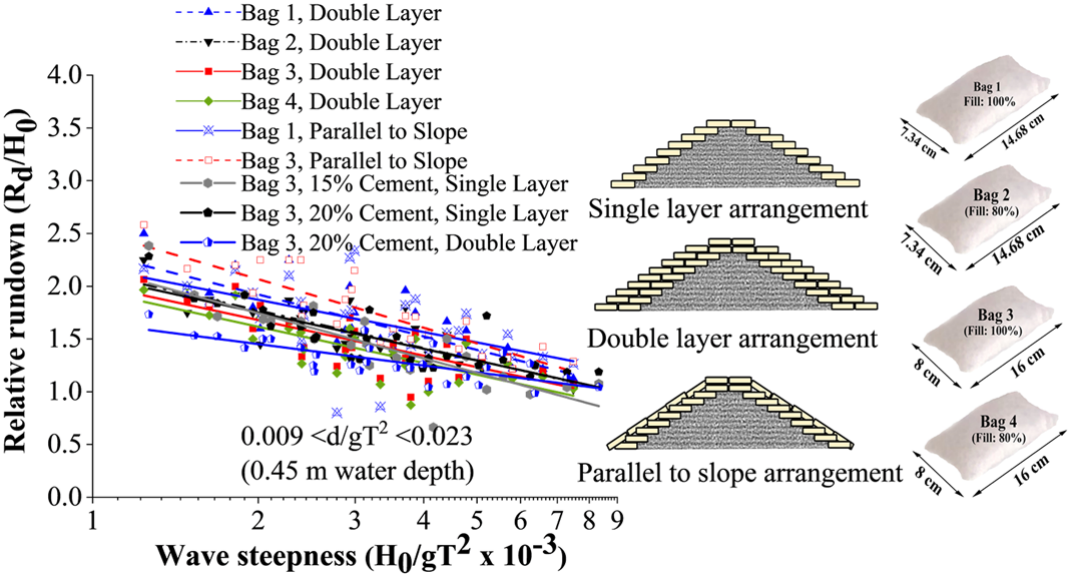
Comparative analysis of rundown behaviour of all the tested configurations.

As far as wave rundown is considered, the slope parallel placements exhibit the maximum value, which ranges up to 2.5 times the incident wave height (see Fig. 19). The structures indicate the least rundown values with 20% cement content (relative rundown ranging from 1.1 to 1.5). The rundown values exhibited by all other models lie between these two cases. The main reason behind high rundown is the continuous covering in slope-parallel placement, reducing the porosity of the structure. As a result, water glides and remains on the structure slope without getting absorbed or dissipated through the pores. Also, 20 to 30% decrease in rundown is exhibited as the size of the armour unit increased. As discussed in the previous sections, increasing the porosity of the structure with increasing armour unit size may be the significant contributing factor to the reduced runup.

#### Comparative analysis of reflection

**Figure 20:**
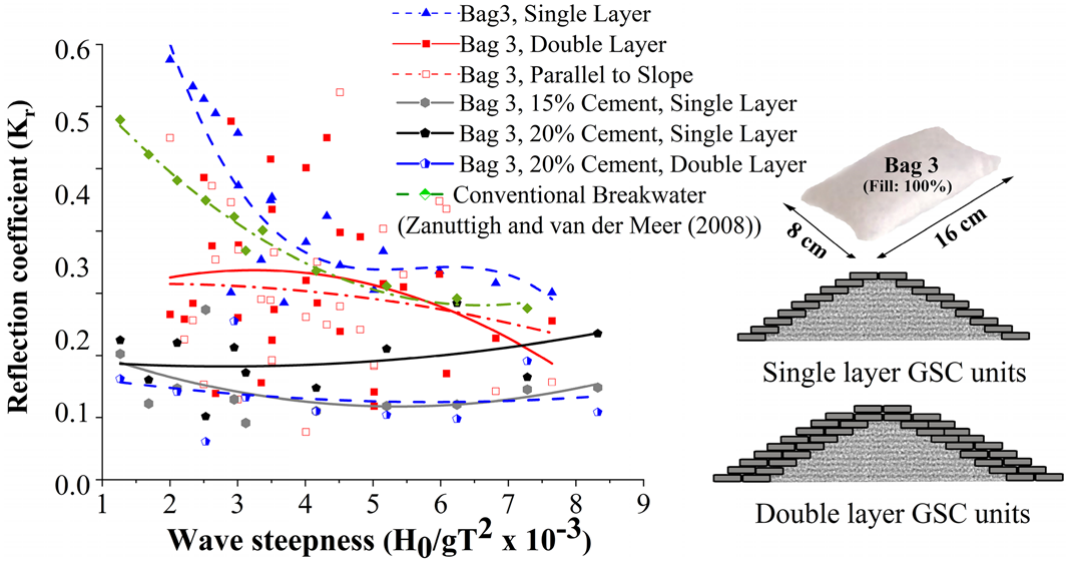
Reflection curves.

The reflection values appear scattered, as illustrated in Fig. 20. One can observe that all other tested configurations exhibit lower reflection rates than the conventional breakwater except GSC single layer configuration. It has to be noted that mortar filled configurations exhibit lower reflection rates, with a maximum reflection coefficient K_r_ of 0.2. Increased porosity, in that case, could be the possible reason for the reduction in reflection.

#### Comparison of stability

**Figure 21:**
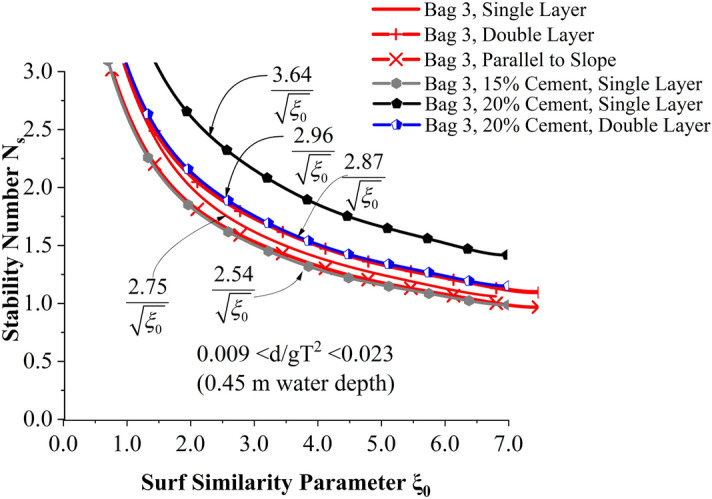
Stability curves for Bag 3 with all the tested arrangements.

Stability curves for all the tested GSC breakwater configurations help analyse the efficacy of various placement techniques. In the present investigation, structure with 'Bag 3’ armour is experimented within all types of arrangements, including single and double layers, slope parallel placements and cement-sand filling. Therefore, stability curves of Bag 3 armour with all the tested arrangements for 0.45 m water depth have been represented in Fig. 21. 0.45 m depth was the most damaging depth among all the experimentation cases, therefore, critical cases of stability tests were only investigated. Cement 20% single layer configuration shows the maximum stability, with slope parallel placements and single layer (15% cement) case being the weakest.

As observed, when GSC units are filled with cement, single-layered structures were up to 22% more stable than double-layer structures. This is contradictory to the fact that stability increases with the number of layers. The possible reason for this instability can be the lack of friction between the layers, as geotextiles are used. Additionally, these GSC units formed pillow-shaped solids (susceptible to easy displacement) with poor interlocking when the cement-sand mixture solidified within the bags. Units filled with sand alone (see “Stability and damage analysis” section) showed lesser stability when compared with cement-filled units. Out of all tested configurations, slope parallel placement showed the least stability due to decreased porosity and increased runup and destabilising activity on structure slope.

## Conclusions

The study aided in analysing the stability and hydraulic performance of mortar-filled GSC breakwaters. On a general note, GSC breakwater is found to perform better when supplemented with cement and sand fill. As a result, this can be suggested for field application as a more vandal-resistant geotextile sand containment system. Each factor affecting the structure's stability as well as hydraulic performance has been extensively reviewed, and the following concluding remarks have been deduced.

1. Of all the tested configurations, breakwater armoured with Bag 3 filled with 15% cement exhibited the maximum wave runup and rundown up to 33% and 31%, respectively.
2. GSC units with 15% cement are observed to have a higher tendency to break within the bag leading to readjustment and closure of surface pores owing to its higher runup ad rundown behaviour.
3. Of all the tested configurations, breakwater structures with singe layer GSC units filled with 20% cement exhibited up to 76% higher wave reflection, with all the tested cases exhibiting lower reflection than conventional breakwaters.
4. When the cement content is increased from 15 to 20%, there is a considerable increase in stability from 13.6 to 43.3%.
5. Single-layer breakwater structure with GSC units containing 20% cement is found to show the maximum stability, while slope parallel placements are the weakest in terms of stability.
6. Similarly, when breakwater structures with GSC units containing 20% cement are altered from a single layer to double layers, a 17.18 to 22.9% decrease in stability is observed.

When the armour units of GSC breakwaters are filled with mortar, up to 43% increased stability is observed with a considerable decrease in wave runup and rundown compared with sand-alone filled GSC breakwaters. As a result, cement-sand filled GSC units can be suggested as a possible alternative to sand alone filled units where vandalism has to be countered. Additionally, the durability and wave resistance of the breakwaters increases when solidified sand units are used. The preliminary investigation on stability and hydraulic performance nomograms can be projected as the major research outcome, as it aids coastal engineers in designing and planning GSC breakwaters. To sum up, out of the three tested configurations, 20% cement-filled bags placed in a single layer exhibited maximum stability and minimum wave runup and rundown. This can be projected as the best performing model as 15% cement-filled single layer structure and 20% cement-filled double layer structure had certain drawbacks. As explained, 15% cement-filled single-layer structure had a tendency to break within the bag resulting in the readjustment of pores, leading to increased runup and rundown. Increased runup and rundown are unadvisable to field practices, as it can lead to wave overtopping and transmission. Additionally, stability is also found to be decreasing. When breakwaters are provided with double layers of mortar-filled units, wave runup and rundown exhibited a favourable decrease while stability was reduced. Thus it is concluded not to suggest double layer placement of solidified GSC units for field applications.

## Data Availability

The datasets used and/or analysed during the current study are available from the corresponding author on reasonable request.
